# P08-05 Community-based interventions to promote physical activity among individuals with social disadvantages

**DOI:** 10.1093/eurpub/ckac095.118

**Published:** 2022-08-29

**Authors:** Simone Kohler, Jana Semrau, Natalie Helsper, Lea Dippon, Karim Abu-Omar, Klaus Pfeifer, Alfred Ruetten

**Affiliations:** Department of Sport Science and Sport, Friedrich-Alexander-University Erlangen-Nuremberg, Erlangen, Germany

**Keywords:** physical activity promotion, community, social disadvantage, inequalities in health

## Abstract

**Background:**

The extent to which people are physically inactive is dependent upon social gradients. Numerous studies have proven that individuals with social disadvantages are not active enough. Parallel to this, several researchers have raised concerns that public health interventions may increase inequalities in the population. However, little is known about the success of community-based physical activity promotion among individuals with social disadvantages. Hence, our goal was to identify the characteristics of successful interventions within this field.

**Methods:**

From March 2015 to March 2019, a search for systematic reviews dealing with community-based physical activity promotion was carried out using the databases PubMed, Scopus, PsycInfo/SPORTDiscus (via Ebscohost), ERIC and IBSS (via ProQuest). Only articles written in English or German were included. Studies without information about socially disadvantaged groups or physical activity promotion in low and middle-income countries were excluded. Checking of the reference lists of included reviews completed the research. Two authors independently conducted the screening, selection and data extraction. Results were synthesized narratively.

**Results:**

In the first step, a total of 2,610 articles were identified. After the screening, 20 publications could be considered, while only six involved individuals with social disadvantages. In particular, these articles described environmental interventions, tailoring, and involvement of the target group, as effective among individuals with social disadvantages. In addition, strategies for gaining political support, intersectionality strategies, and the creation of access routes to reach individuals with social disadvantages were specified as requirements for effectiveness.

**Conclusion:**

The current state of research concerning physical activity interventions in a community setting for individuals with social disadvantages is very limited. Because the target group is so broad, the evidence of successful approaches is heterogeneous. In order to assess the characteristics of physical activity promotion interventions, additional studies that focus on various groups of people with social disadvantages in real-world community settings are needed.

